# *Akkermansia muciniphila* is associated with normal muscle mass and *Eggerthella* is related with sarcopenia in cirrhosis

**DOI:** 10.3389/fnut.2024.1438897

**Published:** 2024-10-30

**Authors:** Irina Efremova, Aliya Alieva, Roman Maslennikov, Elena Poluektova, Maria Zharkova, Anna Kudryavtseva, George Krasnov, Yury Zharikov, Yaroslav Nerestyuk, Anna Karchevskaya, Vladimir Ivashkin

**Affiliations:** ^1^Department of Internal Medicine, Gastroenterology and Hepatology, Sechenov University, Moscow, Russia; ^2^The Interregional Public Organization “Scientific Community for the Promotion of the Clinical Study of the Human Microbiome”, Moscow, Russia; ^3^Post-Genomic Research Laboratory, Engelhardt Institute of Molecular Biology of Russian Academy of Sciences, Moscow, Russia; ^4^Department of Anatomy, Sechenov University, Moscow, Russia; ^5^P. A. Hertsen Moscow Oncology Research Center, Moscow, Russia

**Keywords:** gut-muscle axis, gut microbiome, malnutrition, gut dysbiosis, gut-liver axis

## Abstract

**Background:**

Sarcopenia and gut dysbiosis are common in cirrhosis. The aim is to study the correlations between the gut microbiota taxa and muscle mass level in cirrhosis.

**Methods:**

The study included 40 cirrhosis patients including 18 patients with sarcopenia. The gut microbiota composition was assessed using amplicon sequencing of the hypervariable V3-V4 regions of the 16S rRNA gene. The skeletal muscle mass, subcutaneous and visceral fat levels were assessed with abdominal computed tomography as skeletal muscle, subcutaneous and visceral fat indices (SMI, SFI and VFI).

**Results:**

Patients with sarcopenia had more relative abundance (RA) of Agathobacter, Anaerostipes, Butyricicoccus, Dorea, Eggerthella, Microbacteriaceae, Veillonella and less RA of Akkermansiaceae, *Akkermansia muciniphila*, Verrucomicrobiae and Bilophila compared to patients with normal muscle mass. SMI directly correlated with RA of Akkermansia, *Alistipes indistinctus*, Anaerotruncus, Atopobiaceae, *Bacteroides clarus*, *Bacteroides salyersiae*, Barnesiellaceae, *Bilophila wadsworthia*, Pseudomonadota, Olsenella, and *Parabacteroides distasonis*, and negatively correlated with RA of Anaerostipes and Eggerthella. Sarcopenia was detected in 20.0% patients whose gut microbiota had Akkermansia but not Eggerthella, and in all the patients, whose gut microbiota had Eggerthella but not Akkermansia. The Akkermansia and Eggerthella abundances were independent determinants of SMI. RA of Akkermansia, *Akkermansia muciniphila*, Akkermansiaceae, *Bacteroides salyersiae*, Barnesiella, Bilophila, Desulfobacterota, Verrucomicrobiota and other taxa correlated positively and RA of Anaerovoracaceae, Elusimicrobiaceae, Elusimicrobium, Kiritimatiellae, Spirochaetota, and other taxa correlated negatively with the SFI. RA of Alistripes, Romboutsia, Succinivibrio, and Succinivibrionaceae correlated positively and RA of *Bacteroides thetaiotaomicron* correlated negatively with VFI.

**Conclusion:**

The muscle mass level in cirrhosis correlates with the abundance of several gut microbiota taxa, of which *Akkermansia* and *Eggerthella* seems to be the most important.

## 1 Introduction

Sarcopenia, loss of muscle mass and function, is common in cirrhosis and associated with poor prognosis (1). There are recommendations for the correction of sarcopenia and malnutrition in the latest versions of national and international guidelines for the treatment of cirrhosis (2–6). The mechanism of sarcopenia development in cirrhosis has not been clearly established and seems be multifactorial (1). It is assumed that the gut microbiota plays an important role in this process (1, 7). Recently, systematic (8) and literature (9) reviews has been published on the prospects of the gut microbiota targeted therapy for the treatment and prevention of sarcopenia. Cirrhosis, representing the end of chronic liver diseases, leads to a decrease in its function, the development of stagnation of blood in the intestine and shunting of the blood flowing from it (9). Disorders of the blood circulation in the intestine leads to a slowdown in its clearance, a decrease in the protective function of the intestinal epithelium and an increase in the permeability of the intestinal barrier. All this leads to a change in the composition of the gut microbiota (gut dysbiosis) and an increase in its quantity (small intestinal bacterial overgrowth). An increase in the number of altered microbiota forms a lot of ammonia, which hinders anabolic processes in the muscles (9). A similar effect is exerted by systemic inflammation caused by the penetration of excess altered microbiota through the damaged intestinal barrier into the intestinal wall and further into the portal and systemic circulation (bacterial translocation) (9). These and other disorders of the gut-muscle axis are thought to contribute to the development of sarcopenia in cirrhosis (9).

Gut dysbiosis are associated with a number of complications of cirrhosis (10–12), as well as with poor short-term (13) and long-term (14) prognosis.

The gold standard for sarcopenia assessment is quantitative abdominal CT. The skeletal muscle index (SMI) is calculated as ratio area of total abdominal skeletal muscles at the L3 vertebral level to square of height (2, 15). This method can also assess the area of subcutaneous abdominal and visceral fat at this level. Unlike CT, bioelectrical impedance analysis (BIA) and dual-energy X-ray absorptiometry (DXA) indirectly estimate whole body cell mass, most of which, as a rule, is skeletal muscle tissue (15).

Only 4 papers have been published describing the difference in the composition of the gut microbiota in cirrhosis patients with and without sarcopenia (16–19). However, these studies used different methods for assessing muscle mass, that can be a cause for conflicting results obtained (Table 1).

**Table 1:** Gut microbiota taxa associated with sarcopenia and normal muscle mass in cirrhosis according to published studies.

References	Method for assessing sarcopenia	Methods of statistical analysis	Gut microbiota taxa associated with sarcopenia	Gut microbiota taxa associated with normal muscle mass
(16)	CT	Comparison of a group of patients	*Bacteroides faecis*, *Bacteroides caccae*, *Bacteroides coprocola*, *Escherichia coli*, *Eubacterium infirmum*, *Peptostreptococcus stomatis*, *Weissella* unclassified	*Anaerostipes hadrus*, *Bacteroides uniformis*, *Bifidobacterium catenulatum*, *Clostridium clostridioforme*, *Clostridium asparagiforme*, *Eggerthella* unclassified, *Erysipelotrichaceae* bacteria 2 2 44A and 5 1 63FAA *Granulicella* unclassified, *Lachnospiraceae* bacterium 5 1 63FAA, *Ruminococcus flavefaciens*
(17)	DXA	Comparison of a group of patients, correlation analysis	*Eggerthella*	*Akkermansia*, *Methanobrevibacter*, *Prevotella*
(18)	BIA	Comparison of a group of patients, correlation analysis	*Bacteroidaceae*, *Eggerthella*, *Veillonella*	*Barnesiellaceae*, *Erysipelatoclostridiaceae*, *Anaerotruncus*, *Catenibacterium*, *Coprococcus*, *Desulfovibrio*, *Intestinimonas*, *Oscillospira*, *Senegalimassilia*
(19)	DXA	Comparison of a group of patients	*Micrococcaceae*, *Fusobacterium*, *Rothia*	*Coriobacteriaceae*, *Lachnospiraceae*, *Acidaminococcus*, *Anaerostipes*, *Collinsella*, *Dialister*, *Dorea*, *Megasphaera*, *Ruminococcus*, *Eubacterium hallii*

However, none of the published studies performed a correlation analysis between the abundance of different taxa of the gut microbiota and the muscle mass value assessed with the gold standard (quantitative abdominal CT). Analysis of these correlations is the aim of our study. The information obtained is important, as it will help to identify the key taxa that can be associated for the maintenance of muscle mass in patients with cirrhosis and for its loss. Developing ways to target these taxa could slow or even reverse the development of sarcopenia in cirrhosis.

In addition, this method allows performing a correlation analysis between the level of subcutaneous and visceral fat and the abundance of gut microbiota taxa in cirrhosis, which also has not yet been studied in published studies and became an additional task of the study.

## 2 Materials and methods

### 2.1 Patients

The patients with cirrhosis that were consecutively admitted to the Department of Hepatology of the Clinic for Internal Medicine, Gastroenterology and Hepatology were screened for participation. The procedures were explained to potential participants, and written informed consent was obtained before enrollment. The study was approved by the Ethics Committee of Sechenov University (#04-21 dated 18.02.2021) in accordance with the Declaration of Helsinki.

The inclusion criteria were diagnosis of cirrhosis verified by histological examination or clinical, biochemical, and ultrasound findings, and age between 18 and 70 years. The diagnosis of cirrhosis was established on the basis of presence chronic liver disease, clinical and laboratory findings (presence of signs of portal hypertension and/or decreased liver function), and liver transient elastography data (>12 kPa) (20). The exclusion criteria included the use of lactulose, lactitol, or other prebiotics, probiotics, antibiotics, or metformin in the past 6 wk, alcohol consumption in the past 6 wk, or diagnosis of inflammatory bowel disease, cancer, or any other serious disease. The exclusion criteria were selected to remove the influence of these factors on the composition of the gut microbiota.

### 2.2 Gut microbiome analysis

A stool sample was obtained from each patient and placed in a sterile disposable container the morning after admission and immediately frozen at −80°C.

Total DNA was isolated using the AmpliPrime DNA-sorb-AM kit (NextBio, Moscow, Russia) for clinical specimens, according to the manufacturer’s protocol. The isolated DNA was stored at −20°C. For qualitative and quantitative assessment of the isolated DNA we used NanoDrop 1000 equipment (Thermo Fisher Scientific, Waltham, MA, United States). The 16S library preparation was carried out according to the protocol of 16S Metagenomic Sequencing Library Preparation (Illumina, San Diego, CA, United States), which is recommended for Illumina MiSeq sample prep. The first round of amplification of V3-V4 16S rDNA variable regions was performed using the following primers: forward [341F, CCTACGGGNGGCWGCAG (21)] and reverse (805R, GACTACHVGGGTATCTAATCC). These primers are aimed at the amplification of bacterial (more than 90%) but not archaeal (less than 5%) rRNA genes. The amplification program (Applied Biosystems 2720 Thermal Cycler, Foster City, CA, United States) was as follows: (1) 95°C for 3 min; (2) 30 cycles: 95°C for 30 s; 55°C for 30 s; 72°C for 30 s; (3) 72°C for 5 min; and (4) 4°C.

The derived amplicons were purified using Agencourt AMPure XP (Beckman Coulter, Brea, CA, United States) beads according to the manufacturer’s protocol. The second amplification round was used for double-indexing samples with a combination of specific primers. The amplification program was as follows: (1) 95°C for 3 min; (2) 8 cycles: 95°C for 30 s; 55°C for 30 s; 72°C for 30 s; (3) 72°C for 5 m; and (4) 4°C.

The purification of PCR products was also carried out using Agencourt AMPure XP beads. The concentration of the derived 16S rDNA libraries was measured using a Qubit^®^ 2.0 fluorometer (Invitrogen, Carlsbad, CA, United States) using QuantiT™ dsDNA High-Sensitivity Assay Kit. The purified amplicons were mixed equimolarly according to the derived concentration values. Quality of the libraries was evaluated using an Agilent 2100 Bioanalyzer (Agilent Technologies, Santa Clara, CA, United States) and Agilent DNA 1000 Kit. Sequencing was carried out on a MiSeq machine (Illumina) using the MiSeq Reagent Kit v2 (paired-end reads, 2 × 300 nt).

The data were analyzed as follows. First, forward and reverse reads were merged using MeFiT 1.0, a wrapper for and CASPER 0.8.2 tool (22). The merging was performed with the default MeFiT parameters except for meep-score threshold (0.4) and default CASPER parameters except for minimum overlap (30 bp), threshold for mismatch-ratio (0.5). For the most samples more than 99% reads were successfully merged. Merged amplicons falling within the 420–480 bp range were included in the analysis. For the vast majority of amplicons, the length was 465 bp; for some, 440–460 bp. Next, amplicons were analyzed with the DADA2 1.22 package (a part of the Bioconductor project) for R 4.2.2 (23) in order to remove primers (cutadapt 3.2; primer error rate threshold 0.1, turned on IUPAC), filter reads, correct errors, infer RSV (ribosomal sequence variants) and remove chimeras. Next, taxonomic annotation of the derived RSVs was performed using the naive RDP classifier algorithm (built-in default DADA2 annotation engine) based on Silva (version 138.1) 16S reference sequence database (24). Species assignment was performed only for those RSVs that had 100% homology with reference sequences in the Silva database (version 138.1). Rarefaction curves were calculated using q2-diversity plugin.

### 2.3 Body composition assessment

All patients underwent abdominal CT without contrast enhancement within 1–5 days after hospitalization and stool sample taking. The Myrian software (Intrasense, France) was used for calculation of the total area (in cm^2^) of skeletal muscle, subcutaneous fat, and visceral fat at the level of the L3 vertebra. These results were divided by the square of the height of the patients (in meters) for calculation the skeletal muscle index (SMI), subcutaneous fat index (SFI), and visceral fat index (VFI), respectively. SMI <50 cm^2^/m^2^ in males and < 39 cm^2^/m^2^ in females were used for sarcopenia diagnosis (2).

### 2.4 Statistical analysis

Statistical analysis was performed with STATISTICA 10 (StatSoft Inc., Tulsa, OK, United States). The data are presented as medians [interquartile range]. The significance of the difference between groups was assessed using the Mann–Whitney method. The univariate regression analysis was performed using the Spearman method. Multivariate linear correlation analysis between the abundances of gut microbiota taxa, body composition indices, and the parameters (the patient’s age, gender, and Child-Pugh score value) that significantly correlated with the body composition indices in a univariate regression analysis was performed. To construct this multivariate regression model, we took the abundance of a specific taxon, a specific index, and cirrhosis parameters whose values significantly correlated with this index in univariate linear regression models. *p* values ≤0.05 were considered statistically significant.

An in-depth comparison of the gut microbiota between patients with sarcopenia and normal muscle mass was performed using the Linear discriminant analysis Effect Size (LEfSe).^1^ The functional features of the gut microbiota in patients with sarcopenia and nomal muscle mass were analyzed using the PICRUSt (Phylogenetic Investigation of Communities by Reconstruction of Unobserved States) method (see text footnote 1).

## 3 Results

Of the original 86 patients screened for inclusion, 40 were enrolled in the study and 46 were excluded. We excluded 3 patients in whom the diagnosis of cirrhosis was not confirmed, as well as those who used lactitol (*n* = 24), lactulose (*n* = 6) and antibiotics (*n* = 8) within 6 weeks before inclusion. In addition, 5 patients refused to participate in the study. The main characteristics of the patients are presented in Table 2.

**Table 2:** The main characteristics of the cirrhosis patients.

Parameter	Value
Age, years	51 [42–61]
Male/Female	18/22
Etiology: Alcohol	15
Hepatitis C virus	3
Autoimmune hepatitis	4
Metabolic-associated fatty liver disease	3
Wilson disease	3
Cholestatic liver diseases	6
Cryptogenic	3
Mixed	3
Body mass index, kg/m^2^	27.1 [23.9–29.9]
Skeletal muscle index, cm^2^/m^2^	43.6 [38.3–88.6]
Subcutaneous fat index, cm^2^/m^2^	63.0 [37.2–88.6]
Visceral fat index, cm^2^/m^2^	54.3 [39.3–70.9]
Sarcopenia, n (%)	18 (45.0%)
Red blood cells, 10^12^/L	4.1 [3.6–4.7]
White blood cells, 10^9^/L	5.0 [3.3–6.1]
Platelets, 10^9^/L	106 [75–150]
Serum albumin, g/L	36.8 [33.1–40.6]
Serum total bilirubin, μmol/L	31.9 [18.9–55.5]
Prothrombin (Quick test), %	70 [60–88]
Ascites: grade 0/1/2–3, n	19/14/7
Esophageal varices: 0/1/2–3, n	9/16/15
Hepatic encephalopathy: no/occult/overt, n	27/10/3
Child-Pugh class: A/B/C	12/18/10
Child-Pugh score	8 [6–10]

All samples showed high sequencing depth according to rarefaction curve data.

All studied body composition indices (SMI, SFI, and VFI) directly correlated with BMI. SMI positively correlated with SFI and male gender. SFI correlated positively with all other body composition indexes, age, and female gender, and negatively with cirrhosis severity. VFI positively correlated with SFI and age of patients (Table 3).

**Table 3:** Correlation matrix of indicators of body composition (indices of skeletal muscle [SMI], subcutaneous [SFI] and visceral [VFI] fat), body mass index (BMI), age, gender and Child-Pugh score (CPS) values of included patients.

	SMI	SFI	VFI	BMI	Age	Male gender	CPS
SMI	–	*r* = 0.444; *p* = 0.004	NS	*r* = 0.506; *p* < 0.001	NS	*r* = 0.331; *p* = 0.037	NS
SFI	*r* = 0.444; *p* = 0.004	–	*r* = 0.331; *p* = 0.037	*r* = 0.957; *p* < 0.001	*r* = 0.508; *p* = 0.001	*r* = −0.374; *p* = 0.017	*r* = −0.348; *p* = 0.028
VFI	NS	*r* = 0.331; *p* = 0.037	–	*r* = 0.378; *p* = 0.016	*r* = 0.431; *p* = 0.005	NS	NS

NS, not significant.

**Figure 1:**
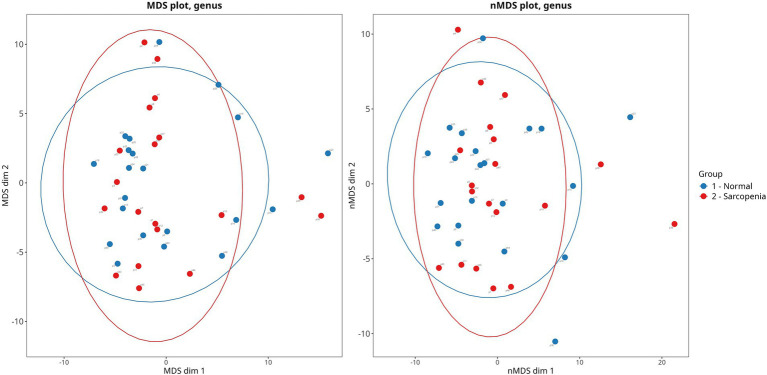
Principal coordinate analysis of gut microbiota beta-diversity (Bray-Curtis method) in cirrhotic patients with sarcopenia and normal muscle mass. PERMANOVA: *p* = 0.761. PERMDISP: *p* = 0.231.

Sarcopenia was detected in 18 patients (45%). These patients had lower SMI and SFI values than patients with normal muscle mass. Except for serum alkaline phosphatase activity, no significant difference in the values of the main indicators of cirrhosis, drugs used, gut microbiota biodiversity, and in the abundance of main gut microbiota taxa (4 phyla most abundantly represented in the gut microbiota, and 3 largest classes within the phylum Bacillota, which is the most abundant phylum of gut microbiota) between these groups (Table 4 and Figure 1). The relative abundance of the major genera and families of the gut microbiota are presented in Supplementary Tables 1, 2.

**Table 4:** The main characteristics of the cirrhotic patients with and without sarcopenia.

Parameter	Sarcopenia (*n* = 18)	Normal muscle mass (*n* = 22)	*p*
Age, years	44 [39–63]	55 [46–61]	0.308
Male/Female	9/9	9/13	0.399
Body mass index, kg/m^2^	24.0 [21.3–26.8]	29.7 [27.1–33.0]	<0.001
Skeletal muscle index, cm^2^/m^2^	36.9 [33.8–40.2]	52.3 [44.3–58.5]	<0.001
Subcutaneous fat index, cm^2^/m^2^	37.6 [33.8–40.2]	87.1 [62.7–123]	<0.001
Visceral fat index, cm^2^/m^2^	47.1 [36.5–65.6]	60 [40–82]	0.161
Serum ALT, U/L	47 [25–69]	37 [23–72]	0.438
Serum AST, U/L	58 [41–74]	44 [33–89]	0.438
Serum alkaline phosphatase, U/L	296 [233–715]	240 [173–308]	0.029
Serum GGT, U/L	94 [48–147]	42 [29–152]	0.157
Serum albumin, g/L	35.3 [33.1–37.7]	37.3 [33.2–42.9]	0.226
Serum total bilirubin, μmol/L	32.6 [21.-72.4]	27.7 [18.5–47.7]	0.321
Prothrombin (Quick test), %	71 [60–92]	70 [60–86]	0.817
Platelet, 10^9^/L	114 [72–166]	104 [78–125]	0.946
Ascites: grade 0–1/2–3, n	14/4	19/3	0.383
Esophageal varices: 0–1/2–3, n	10/8	15/7	0.311
Hepatic encephalopathy: no/present, n	12/6	15/7	0.592
Child-Pugh class: A/B + C	3/15	9/13	0.093
Child-Pugh score	8 [7–9]	7 [6–10]	0.463
Bristol stool scale	4 [4–4]	4 [3–4]	0.682
Gut microbiota biodiversity			
Chao 1 index	389 [238–454]	348 [258–545]	0.903
ACE	346 [238–443]	347 [254–541]	0.924
Shannon index	4.12 [3.01–4.29]	3.56 [3.22–4.42]	0.881
Shannon effective number	62 [20–73]	35 [25–83]	0.881
Total count of reads, 10^3^	54.9 [42.2–61.7]	57.0 [45.5–66.9]	0.396
Relative abundance of main gut microbiota taxa			
Bacillota	41.1 [28.6–57.0]	35.2 [27.1–52.8]	0.334
Pseudomonadota	3.8 [1.4–9.3]	5.5 [2.3–8.5]	0.362
Bacteroidota	38.7 [29.1–58.5]	40.8 [20.2–48.9]	0.881
Actinomycetota	0.8 [0.2–3.9]	1.1 [0.2–3.3]	0.924
Bacilli	0.4 [0.2–1.3]	0.4 [0.2–1.1]	0.946
Clostridia	38.3 [28.0–53.5]	31.9 [21.8–51.2]	0.456
Negativicutes	1.8 [0.6–3.1]	1.3 [0.5–3.6]	0.596

Serum alkaline phosphatase activity did not significantly correlate with SMI (*p* = 0.221) and VFI (*p* = 0.349). However it was inversely correlated with SFI (*r* = −0.481; *p* = 0.002), which provided a significant difference in the value of this enzyme between patients with sarcopenia and with normal muscle mass.

**Figure 2:**
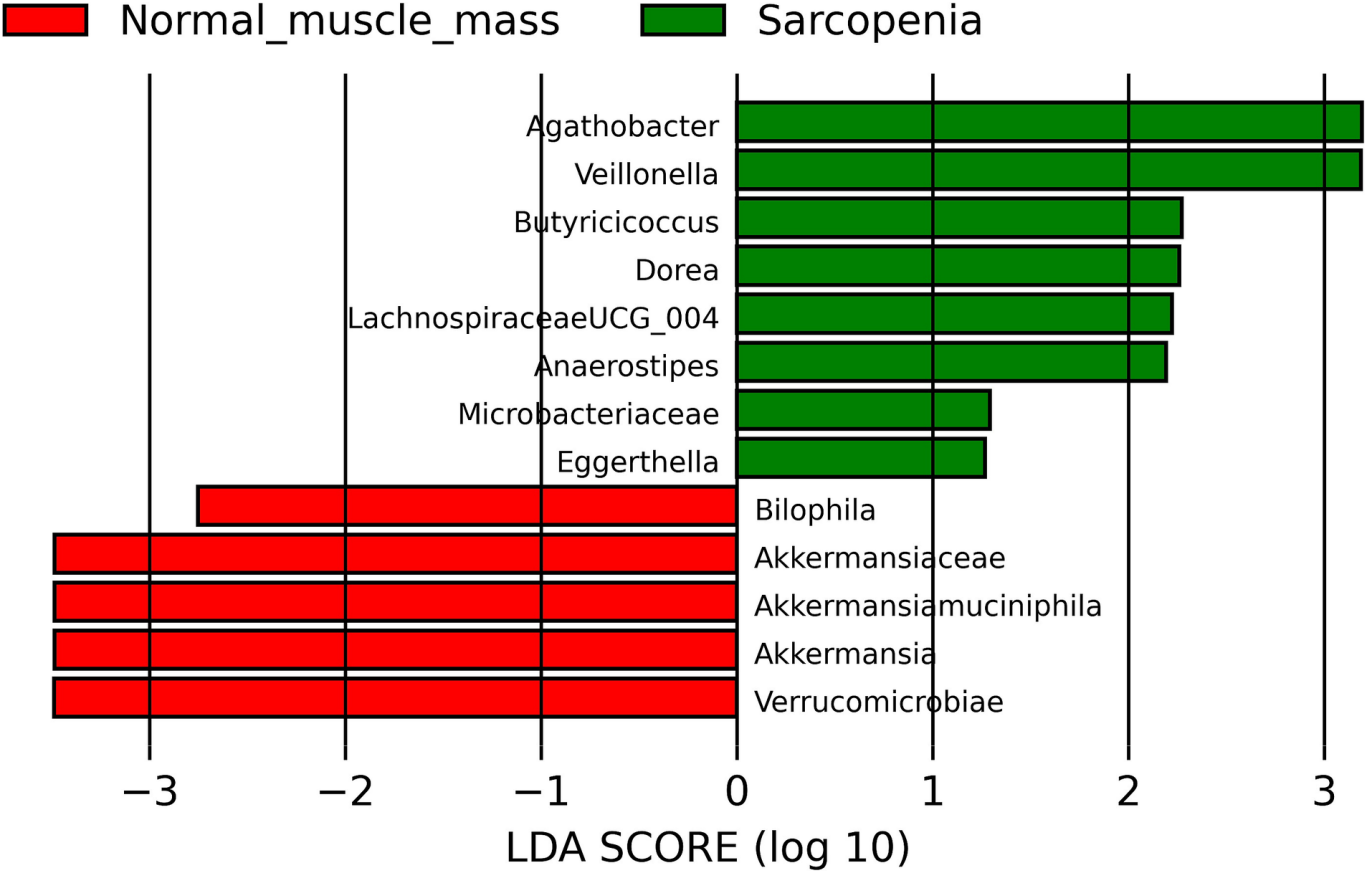
Linear discriminant analysis Effect Size (LEfSe) of comparison of the gut microbiota of cirrhotic patients with sarcopenia and normal muscle mass.

Patients with sarcopenia had more abundance of *Agathobacter*, *Anaerostipes*, *Butyricicoccus*, *Dorea*, *Eggerthella*, *Lachnospiraceae UCG_004*, *Microbacteriaceae*, *Veillonella* and less abundance of *Akkermansiaceae*, *Akkermansia*, *Akkermansia muciniphila*, Verrucomicrobiota (phylum included *Akkermansiaceae* family) and *Bilophila* compared to patients with normal muscle mass (Figure 2).

Phylogenetic Investigation of Communities by Reconstruction of Unobserved States (PICRUSt) revealed that gut microbiota of cirrhosis patients with sarcopenia had more abundance of genes involved in lipopolysaccharide biosynthesis (*p* = 0.023), carbohydrate digestion and absorption (*p* = 0.019), folate biosynthesis (*p* = 0.038), and citrate cycle (*p* = 0.033), but less abundance of genes involved in biosynthesis of antibiotics (*p* = 0.021), biofilm formation (*p* = 0.031), and pentose phosphate pathway (*p* = 0.038) in comparison with cirrhosis patients with normal muscle mass.

According to the results of multivariate regression analysis, the level of muscle mass (SMI) directly correlated with the abundance of *Akkermansia muciniphila*, *Alistipes indistinctus*, *Anaerotruncus colihominis*, *Bacteroides clarus*, *Bacteroides salyersiae*, *Barnesiellaceae*, *Bilophila*, *Desulfovibrionaceae*, *Parabacteroides distasonis*, and Verrucomicrobiota and other taxa, and negatively correlated with the abundance of *Anaerostripes* and *Eggerthella* (Table 5).

**Table 5:** Significant correlations (*r*-value) of skeletal muscle, subcutanious and visceral fat indicis with gut microbiota taxa in patients with cirrhosis by multivariate linear correlation analysis (adjusted for confounders).

Gut microbiota taxon	Skeletal muscle index	Subcutaneous fat index	Visceral fat index
*Akkermansia*	0.469	0.431	
*Akkermansia muciniphila*	0.460	0.420	
*Akkermansiaceae*	0.469	0.431	
*Alistipes*			0.298
*Alistipes indistinctus*	0.420	0.396	
*Anaerostipes*	−0.298		
*Anaerotruncus colihominis*	0.313	0.335	
*Anaerovoracaceae*		−0.382	
*Atopobiaceae*	0.388	0.456	
*Bacteroides clarus*	0.309		
*Bacteroides salyersiae*	0.418	0.441	
*Bacteroides thetaiotaomicron*			−0.301
*Barnesiella*	0.442	0.470	
*Barnesiella intestinihominis*	0.405	0.458	
*Barnesiellaceae*	0.446	0.473	
*Bilophila*	0.432	0.485	
*Bilophila wadsworthia*	0.399	0.464	
*Desulfovibrionaceae*	0.392	0.436	
Deltaproteobacteria	0.392	0.436	
*Eggerthella*	−0.324		
*Elusimicrobia*		−0.343	
*Elusimicrobiaceae*		−0.347	
*Elusimicrobium*		−0.344	
*Kiritimatiellia*		−0.347	
*Olsenella*	0.340	0.394	
*Parabacteroides distasonis*	0.287	0.344	
*Romboutsia*			0.310
*Spirochaetaceae*		−0.344	
Spirochaetia		−0.347	
Spirochaetota		−0.344	
*Succinivibrio*			0.386
*Succinivibrionaceae*			0.360
*Treponema*		−0.347	
Verrucomicrobiota	0.448	0.454	

Color scale				
*p*-value	<0.010	0.010–0.025	0.026–0.050	>0.050

*Akkermansia* was detected in the gut microbiota in 27.8% of patients with sarcopenia and in 63.7% of patients with normal muscle mass *p* = 0.025. *Eggerthella* was detected in the gut microbiota in 44.4% of patients with sarcopenia and in 9.1% of patients with normal muscle mass (*p* = 0.013). Sarcopenia was detected in 20.0% of patients whose gut microbiota had *Akkermansia* and no *Eggerthella* detected, and in all the patients whose gut microbiota had *Eggertella* and no *Akkermansia* detected (*p* = 0.002). Multivariate regression analysis showed that the abundances of *Akkermansia* and *Eggerthella* were independent determinants of SMI (*r* = 0.372, *p* = 0.013; and *r* = −0.303, *p* = 0.041, respectively).

The abundance of *Akkermansia*, *Akkermansia muciniphila*, *Akkermansiaceae*, *Bacteroides salyersiae*, *Barnesiella*, *Bilophila*, Desulfobacterota, Verrucomicrobiota and other taxa correlated positively and the abundance of *Anaerovoracaceae*, *Elusimicrobiaceae*, *Elusimicrobium*, *Kiritimatiellae*, Spirochaetota, and other taxa correlated negatively with the amount of subcutaneous fat (SFI) according to the results of multivariate analysis (Table 5).

The abundance of *Alistipes*, *Romboutsia*, *Succinivibrio*, and *Succinivibrionaceae* correlated positively and the abundance of *Bacteroides thetaiotaomicron* correlated negatively with the amount of visceral fat (VFI) according to the results of multivariate analysis (Table 5).

Abundance of none of the above taxa was significantly correlated with Bristol stool scale scores.

**Figure 3:**
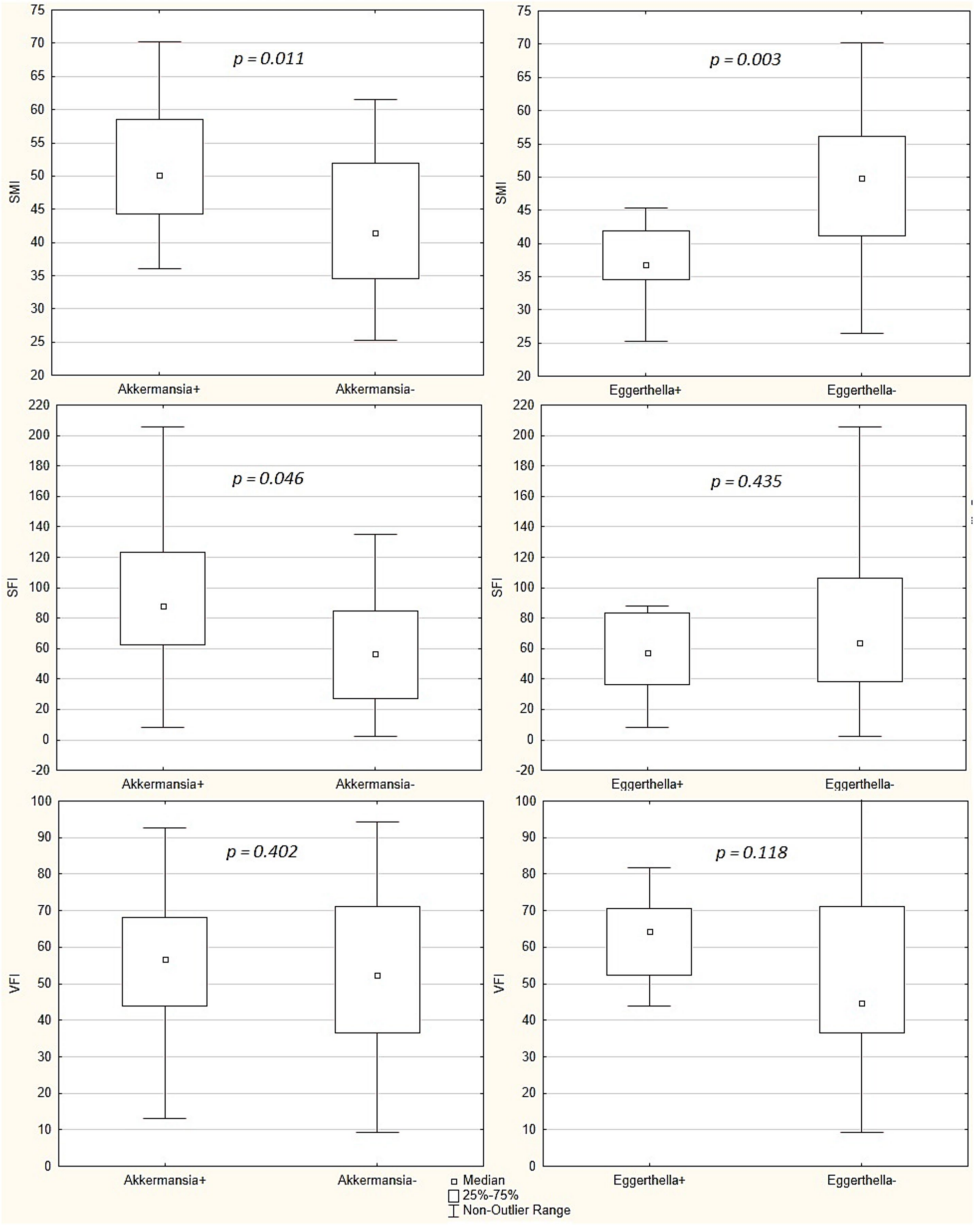
Comparison of skeletal mass index (SMI), subcutaneous fat index (SFI) and visceral fat index (VFI) between patients whose gut microbiota had *Akkermansia municiphila* detected and patients whose gut microbiota had not these bacteria detected (“Akkermansia+” group [*n* = 19] vs. “Akkermansia-“[*n* = 21] group, respectively), and between patients whose gut microbiota had *Eggerthella* detected and patients whose gut microbiota had not these bacteria detected (“Eggerthella+” group [*n* = 11] vs. “Eggerthella-“group [*n* = 29], respectively). Middle point indicates the median, the box values show the interquartile range, and the whiskers display the non-outlier range. The Mann–Whitney test was used.

In patients in whose gut microbiota *Akkermansia muciniphila* were detected, SMI and SFI were higher than in those in whom these bacteria were not detected. There was no significant difference in VFI value between these groups of patients. In patients in whose gut microbiota *Eggerthella* were detected, SMI were lower than in those in whom these bacteria were not detected. There was no significant difference in SFI and VFI values between these groups of patients (Figure 3).

## 4 Discussion

Cirrhosis is the final stage of chronic liver diseases. However, cirrhosis is not limited to damage to the liver, it also leads to disorders in other organs: the gut, brain, hematopoietic system, lungs, kidneys, muscles, and so on. Despite extensive study, the mechanism for the development of these cirrhosis manifestations has not yet been established clearly. Recently, much attention has been paid to the gut microbiota. The changes in the composition of microbiota (gut dysbiosis) in cirrhosis consist of an increase in the proportion of facultative anaerobes from the Bacilli class and the Proteobacteria phylum and the proportion of Proteobacteria bearing active endotoxin, a decrease in the proportion of beneficial obligate anaerobes from the Clostridia class, which includes *Ruminococcaceae* and *Lachnospiraceae*, and other changes (9, 10).

The concept of the gut-liver axis was proposed, which explained the role of gut dysbiosis in the development and aggravation of portal hypertension, one of the main manifestations of cirrhosis, through a sequence of events: an increase in the composition of the gut microbiota the abundance of facultative anaerobes capable of cell bacterial translocation (Bacilli and Proteobacteria) and the abundance of producers of active endotoxin (Proteobacteria) - > bacterial translocation (cellular and molecular [endotoxin]) - > systemic inflammation - > systemic vasodilation - > hyperdynamic circulation - > increased mesenteric blood flow - > development and aggravation of portal hypertension (9, 10).

By analogy, it was suggested that gut dysbiosis may play an important role in the development of other manifestations of cirrhosis, including sarcopenia. However, the published results do not yet give a clear idea of the specific mechanisms of these relationships. Analysis of Table 1, which presents the results of 4 published studies, shows their pronounced heterogeneity (16–19).

It should be noted that neither our study nor those ones showed an association between sarcopenia and the main taxa of the gut microbiota, the change in which is the above-described cirrhosis-associated gut dysbiosis. As can be seen from Supplementary Tables 1, 2, the gut microbiota taxa that significantly correlated with skeletal muscle and fat mass indices were minor, suggesting the existence of more subtle mechanisms of the gut-muscle axis in cirrhosis.

The part of the taxa that were associated with normal muscle mass in our study [*Akkermansia* (17), *Barnesiellaceae* (18), and Desulfobacterota (18)] was similar to the analog results of the previous studies. At the same time, other taxa (*Alistipes indistinctus*, *Anaerotruncus colihominis*, *Atopobiaceae*, *Bacteroides clarus*, *Bacteroides salyersiae*, *Bilophila*, *Olsenella*, and *Parabacteroides distasonis*) were not previously described in association with normal muscle mass in cirrhosis. *Eggerthella* were associated with a decrease in muscle mass in our study and this matched with previous studies (17, 18). *Anaerostipes* were associated with sarcopenia in our study and with normal muscle mass in the other studies (16, 19).

Such inconsistency can be explained by different methods of assessing muscle mass, the ethnic characteristics of the participants and other facts. Estimating muscle mass in cirrhosis is indeed an important problem. Of all the proposed methods, only quantitative CT and MRI allow direct assessment of muscle mass, but only in one section. Quantitative CT and MRI of the whole body are the ideal methods for these, but these techniques have not yet been widely adopted. Neither BIA nor DXA estimate muscle mass directly and this can be a source of biases, despite the fact that these methods are recommended for this purpose in clinical practice (2). It seems more correct in scientific practice to evaluate the muscle mass by a direct method (CT or MRI), although these methods also carry the risk of biases in transferring the state of the muscles of one test zone to the entire body. The differences in the methods of gut microbiota collection, storage and analysis can also be sources of biases.

Of all the bacteria, the abundance of which directly correlates with muscle mass in cirrhosis in our study, *Akkermansia muciniphila* from the *Akkermansiaceae* family of the Verrucomicrobiota phylum attracts the most attention. These bacteria showed the ability to strengthen the intestinal barrier (25), which prevents bacterial translocation and the development of systemic inflammation, which is believed to play an important role in the development of sarcopenia in cirrhosis [1; 10]. The protective effect of dietary fiber intake against cancer-associated sarcopenia in an animal experiment was associated with an increase in the abundance of *Akkermansia* in the gut microbiota (26). An important role of *Akkermansia* in realizing the effect of long-term exercise on muscle mass growth has been shown in other animal study (27). Other animal studies also showed a correlation of the positive effect on muscle mass and muscle function of various dietary interventions with an increase in the abundance of *Akkermansia* in the gut microbiota (28–33). Oral administration of *Akkermansia* increased muscle mass in aged mice (34), reduced endoplasmic reticulum stress in muscle, decreased plasma levels of lipopolysaccharide-binding protein and inflammatory signaling in muscle in the lean chow diet-fed mice (35).

Other interesting gut microbiota taxa that was associated with the changes in muscle mass in cirrhosis in our and other studies is *Eggerthella*. These bacteria activate the cytokine pathway of Th17 cells involved in the pathogenesis of inflammatory bowel diseases (36), participates in the biotransformation of bile acids (37) and in the formation of uremic toxins (37), associated with a decrease in muscle mass in previous studies (17, 18, 38, 39) and osteoporosis (40). High levels of *Eggerthella* in the maternal intestines are associated with smaller weight and head diameter of infant (41). Obese patients, on the contrary, had a lower level of these bacteria in the gut (42). Based on the foregoing, it can be assumed that these bacteria, by activating inflammatory processes in the intestine, are able to enhance bacterial translocation and the systemic inflammation caused by it, The systemic inflammation is characterized by a catabolic status, contributing to the development of sarcopenia (1, 9).

Based on this, we suggest that the insufficient abundance of *Akkermansia* and the excess abundance of *Eggerthella* in the gut microbiota may play important roles in the development of sarcopenia in cirrhosis. The role of other taxa correlated with muscle mass in patients with cirrhosis in our study is much less studied and seems to be much less significant.

We showed a good correlation between the amount of subcutaneous fat and muscle mass in patients with cirrhosis, which may indicate the presence of common ways to reduce the amount of subcutaneous adipose and muscle tissues, that is, general malnutrition in cirrhosis. None of our patients had sarcopenic obesity, that is, a combination of obesity and sarcopenia, which makes this condition rare in cirrhosis.

We also performed a correlation analysis between gut microbiota taxa and visceral fat level that is a marker of “bad” abdominal obesity. Among the bacteria associated with a higher proportion of visceral fat, the most interesting is *Romboutsia*, the abundance of which was increased in the gut microbiota of mice with experimental metabolic-associated fatty liver disease (43), in the development of which visceral fat is of great importance. The gut level of these bacteria was associated with obesity in human (44, 45). Further studies on the differential association of the abundance of gut microbiota taxa with the level of visceral and subcutaneous fat are required to clarify the pathogenesis of visceral (“bad”) and somatic (“neutral”) obesity, including in patients with liver diseases.

The associations identified are of clinical significance, as they suggest a new application point for drugs in the treatment of sarcopenia in cirrhosis. Drugs based on live or inactivated *Akkermansia* could prevent or even reverse the development of this cirrhotic complication. RCTs are needed. The same applies to drugs, such as specific phages, that can suppress the growth of *Eggerthella*. However, testing the clinical application of the obtained results remains a challenge for future researchers.

It should be emphasized, however, that we found associations, not causations, so these results should be interpreted with caution.

PICRUSt analysis showed that the gut microbiota of sarcopenic patients had more genes involved in lipopolysaccharide production, which may predispose them to more severe endotoxemia and systemic inflammation, which is associated with sarcopenia in the general population. In addition, this microbiota had a higher representation of genes for carbohydrate digestion, which may predispose them to greater competition with the host for these nutrients and also contribute to the development of sarcopenia. Moreover, it had fewer genes for antibiotic production, which may predispose it to bacterial overgrowth and their greater competition with the host for nutrients.

The strength of our study is that it was the first in cirrhotic patients to conduct a correlation analysis between the gut microbiota taxa and muscle mass level, which was assessed using the gold standard method. It was also the first to perform a correlation analysis the gut microbiota taxa with visceral fat level in cirrhotic patients.

A limitation of our study is small number of included patients, which, however, did not prevent us from obtaining significant correlations. Another limitation of our study is that we were unfortunately unable to analyze serum for biomarkers of intestinal permeability, bacterial translocation, myostatin, ammonia, bile acids, and others that are thought to be involved in the gut-muscle axis in cirrhosis. This remains a challenge for future studies.

## 5 Conclusion

The level of muscle mass in cirrhosis correlates with the abundance of a number of gut microbiota taxa, of which *Akkermansia* and *Eggerthella* seems to be the most important. Larger studies, including the use of drugs that affect the abundances of *Akkermansia* and *Eggerthella* in the gut microbiota, are required to test this hypothesis.

## Data Availability

The raw data supporting the conclusions of this article will be made available by the authors, without undue reservation.

## Footnotes

^1^online resource “http://galaxy.biobakery.org/” was used
