# Supplementary material for: Akkermansia muciniphila is associated with normal muscle mass and Eggerthella is related with sarcopenia in cirrhosis
Source: Front Nutr. 2024 Oct 30;11:1438897. doi: 10.3389/fnut.2024.1438897 (PMC11557486; doi:10.3389/fnut.2024.1438897)
Supplement: Supplementary file 1 [file Table_1.DOCX]

Supplementary Table 1. The main gut microbiota genera in cirrhosis patients with sarcopenia and normal muscle mass (relative abundance, %)

| Gut microbiota genera | Sarcopenia (n = 18) | Normal muscle mass (n = 22) |
| --- | --- | --- |
| Agathobacter | 1.63[0.41-3.65] | 0.5[0.2-1.95] |
| Akkermansia | 0[0-0] | 0.02[0-0.46] |
| Alistipes | 0.85[0.08-2.12] | 1.25[0.14-2.72] |
| Allisonella | 0[0-0] | 0[0-0.01] |
| Alloprevotella | 0[0-0.23] | 0[0-0] |
| Anaerostipes | 0.07[0.01-0.31] | 0.01[0-0.06] |
| Anaerotruncus | 0[0-0.01] | 0[0-0.04] |
| Bacillus | 0.01[0-0.03] | 0.01[0-0.02] |
| Bacteroides | 9.02[1.44-22.74] | 5.84[3.45-37.41] |
| Barnesiella | 0.03[0-0.18] | 0.02[0-0.25] |
| Bifidobacterium | 0.34[0.09-2.99] | 0.61[0.09-3] |
| Bilophila | 0.04[0-0.18] | 0.23[0.05-0.89] |
| Blautia | 1.00[0.31-1.79] | 0.27[0.17-0.69] |
| Brucella | 0.01[0-0.03] | 0.02[0-0.03] |
| Butyricicoccus | 0.14[0.06-0.33] | 0.08[0.03-0.11] |
| Butyricimonas | 0.17[0-0.7] | 0.18[0-0.63] |
| Campylobacter | 0[0-0.01] | 0[0-0] |
| Caproiciproducens | 0[0-0.02] | 0[0-0.02] |
| Catenibacterium | 0[0-0.05] | 0[0-0.19] |
| Christensenellaceae R-7 group | 0.23[0-1.67] | 0.35[0-2.58] |
| Clostridium sensu stricto 1 | 0.02[0-0.05] | 0.01[0-0.32] |
| Colidextribacter | 0.17[0.08-0.4] | 0.16[0.06-0.25] |
| Collinsella | 0.06[0-0.21] | 0.05[0.03-0.09] |
| Coprobacter | 0[0-0.01] | 0.01[0-0.08] |
| Coprococcus | 0.38[0.18-0.9] | 0.71[0.12-1.21] |
| Desulfovibrio | 0[0-0.32] | 0.01[0-0.36] |
| Dialister | 0.06[0-1.09] | 0.38[0-1.89] |
| Dorea | 0.23[0.11-0.39] | 0.12[0.07-0.18] |
| Eggerthella | 0[0-0.01] | 0[0-0] |
| Eisenbergiella | 0[0-0] | 0[0-0.01] |
| Erysipelatoclostridium | 0[0-0.06] | 0[0-0] |
| Erysipelotrichaceae UCG-003 | 0.02[0-0.08] | 0.01[0-0.04] |
| Escherichia/Shigella | 0.41[0.14-1.97] | 0.49[0.05-3.5] |
| Faecalibacterium | 6.58[2.67-14.23] | 3.85[1.92-8.8] |
| Flavonifractor | 0.09[0.06-0.3] | 0.06[0.03-0.23] |
| Fournierella | 0[0-0] | 0[0-0.02] |
| Fusicatenibacter | 0.09[0.01-0.29] | 0.05[0.02-0.16] |
| Fusobacterium | 0[0-0] | 0[0-0.01] |
| Haemophilus | 0.01[0-0.06] | 0[0-0.01] |
| Herbaspirillum | 0.05[0.01-0.13] | 0.04[0.01-0.07] |
| Holdemanella | 0.01[0-0.03] | 0[0-0.03] |
| Hungatella | 0.01[0-0.05] | 0[0-0.01] |
| Intestinibacter | 0[0-0.04] | 0[0-0] |
| Intestinimonas | 0.03[0-0.05] | 0.02[0-0.07] |
| Klebsiella | 0[0-0.02] | 0[0-0.03] |
| Lachnoclostridium | 0.57[0.24-1.27] | 0.54[0.25-1.31] |
| Lachnospira | 0.8[0.21-1.32] | 0.64[0.07-1.04] |
| Lactobacillus | 0[0-0.04] | 0[0-0.05] |
| Marvinbryantia | 0[0-0.01] | 0[0-0.01] |
| Megamonas | 0[0-0] | 0[0-0] |
| Megasphaera | 0[0-0] | 0[0-0.08] |
| Mitsuokella | 0[0-0] | 0[0-0] |
| Monoglobus | 0.03[0-0.16] | 0.05[0-0.14] |
| Negativibacillus | 0[0-0] | 0[0-0] |
| Ochrobactrum | 0[0-0.02] | 0[0-0.02] |
| Odoribacter | 0.08[0-0.17] | 0.11[0.03-0.17] |
| Olsenella | 0[0-0] | 0[0-0.01] |
| Oscillibacter | 0.39[0.13-1.05] | 0.3[0.16-0.82] |
| Oscillospira | 0.01[0-0.03] | 0.01[0-0.04] |
| Paludicola | 0[0-0] | 0[0-0.01] |
| Parabacteroides | 0.45[0.12-1.02] | 0.75[0.22-2.22] |
| Paraprevotella | 0.02[0-0.56] | 0.01[0-0.26] |
| Parasutterella | 0[0-0.01] | 0[0-0.03] |
| Phascolarctobacterium | 0.26[0-0.49] | 0.04[0-0.27] |
| Prevotella | 8.14[0.02-23.52] | 2.54[0.03-25.35] |
| Pseudocitrobacter | 0.01[0-0.01] | 0[0-0.02] |
| Pseudomonas | 0.06[0.02-0.17] | 0.05[0.02-0.1] |
| Pyramidobacter | 0[0-0] | 0[0-0] |
| Romboutsia | 0[0-0.02] | 0[0-0.02] |
| Roseburia | 0.67[0.42-1.4] | 0.53[0.02-1.27] |
| Ruminococcus | 0.26[0.07-1.22] | 0.58[0.03-1.48] |
| Senegalimassilia | 0[0-0.02] | 0[0-0.01] |
| Serratia | 0[0-0.03] | 0[0-0.02] |
| Slackia | 0[0-0.03] | 0[0-0.01] |
| Sphingomonas | 0.06[0.01-0.19] | 0.05[0.02-0.1] |
| Stenotrophomonas | 0.04[0.01-0.07] | 0.02[0-0.04] |
| Streptococcus | 0.04[0.01-0.31] | 0.03[0.01-0.12] |
| Subdoligranulum | 0.67[0.21-1.14] | 0.86[0.14-1.68] |
| Succinivibrio | 0[0-0] | 0[0-0] |
| Sutterella | 0.47[0-1.12] | 1.35[0.45-3.13] |
| Veillonella | 0.19[0.01-0.31] | 0.01[0-0.07] |
| Victivallis | 0[0-0] | 0[0-0.02] |

Supplementary Table 2. The main gut microbiota families in cirrhosis patients with sarcopenia and normal muscle mass (relative abundance, %)

| Gut microbiota families | Sarcopenia (n = 18) | Normal muscle mass (n = 22) |
| --- | --- | --- |
| Acidaminococcaceae | 0,26[0-0,6] | 0,13[0-0,72] |
| Akkermansiaceae | 0[0-0] | 0,02[0-0,46] |
| Alcaligenaceae | 0[0-0,02] | 0[0-0,01] |
| Anaerovoracaceae | 0,03[0,01-0,1] | 0,04[0-0,06] |
| Atopobiaceae | 0[0-0,01] | 0[0-0,02] |
| Bacillaceae | 0,01[0-0,03] | 0,01[0-0,02] |
| Bacteroidaceae | 9,01[1,44-22,73] | 5,84[3,45-37,4] |
| Barnesiellaceae | 0,04[0-0,18] | 0,11[0,01-0,27] |
| Bifidobacteriaceae | 0,35[0,09-2,99] | 0,62[0,09-3,1] |
| Butyricicoccaceae | 0,16[0,07-0,35] | 0,1[0,04-0,16] |
| Campylobacteraceae | 0[0-0,01] | 0[0-0] |
| Caulobacteraceae | 0[0-0,01] | 0[0-0,01] |
| Christensenellaceae | 0,23[0-1,68] | 0,37[0-2,6] |
| Clostridiaceae | 0,02[0-0,05] | 0,02[0-0,33] |
| Coriobacteriaceae | 0,06[0-0,21] | 0,05[0,03-0,09] |
| Desulfovibrionaceae | 0,28[0-0,45] | 0,45[0,19-1,26] |
| Enterobacteriaceae | 1,01[0,2-2,92] | 1,27[0,23-5,06] |
| Erysipelatoclostridiaceae | 0,1[0,08-0,23] | 0,05[0,02-0,2] |
| Erysipelotrichaceae | 0,03[0,01-0,12] | 0,03[0-0,09] |
| Fusobacteriaceae | 0[0-0] | 0[0-0,01] |
| Lachnospiraceae | 14,04[6,1-17,78] | 9,62[5,94-12,97] |
| Lactobacillaceae | 0[0-0,04] | 0[0-0,05] |
| Marinifilaceae | 0,42[0,07-0,82] | 0,26[0,1-0,89] |
| Microbacteriaceae | 0[0-0,01] | 0[0-0] |
| Monoglobaceae | 0,03[0-0,16] | 0,05[0-0,14] |
| Oscillospiraceae | 6,44[4,05-10,31] | 5,68[3,64-9,45] |
| Oxalobacteraceae | 0,06[0,02-0,14] | 0,05[0,02-0,08] |
| Pasteurellaceae | 0,01[0-0,06] | 0[0-0,01] |
| Peptococcaceae | 0[0-0,01] | 0[0-0,01] |
| Peptostreptococcaceae | 0,02[0-0,08] | 0,01[0-0,02] |
| Prevotellaceae | 11,2[0,97-32,07] | 2,71[0,26-27,98] |
| Pseudomonadaceae | 0,06[0,02-0,18] | 0,05[0,02-0,1] |
| Rhizobiaceae | 0,02[0,01-0,07] | 0,03[0,01-0,05] |
| Rikenellaceae | 1,95[0,33-4,2] | 1,71[0,43-3,15] |
| Ruminococcaceae | 11,54[4,34-18,98] | 6,09[4,3-13,34] |
| Sphingomonadaceae | 0,07[0,01-0,19] | 0,05[0,02-0,1] |
| Streptococcaceae | 0,04[0,02-0,33] | 0,04[0,01-0,12] |
| Sutterellaceae | 0,75[0,24-1,5] | 1,39[0,56-3,12] |
| Synergistaceae | 0[0-0] | 0[0-0,01] |
| Tannerellaceae | 0,45[0,12-1,23] | 0,74[0,22-2,24] |
| Veillonellaceae | 0,51[0,13-2,45] | 1,35[0,05-2,23] |
| Xanthomonadaceae | 0,04[0,01-0,07] | 0,02[0-0,04] |
| Yersiniaceae | 0,01[0-0,03] | 0[0-0,02] |
